# Acute Forearm and Hand Compartment Syndrome in a Child Following Delayed Presentation of Forearm Trauma: A Case Report and Literature Review

**DOI:** 10.3390/reports9020180

**Published:** 2026-06-10

**Authors:** Abdulmonem Alsiddiky, Mishari Alanezi, Nouf Alabdulkarim, Bandar Aljammaz, Othman Alabdullah, Saad Alkahtani, Razan Alshatwi, Abdulrahman Alrajhi

**Affiliations:** 1 Professor of Orthopedics, Director of Research Chair of Spinal Deformities, Department of Orthopedics, College of Medicine, King Saud University, Riyadh 12372, Saudi Arabia; 2 College of Medicine, King Saud University, Riyadh 12372, Saudi Arabia; 3 Department of Orthopedic Surgery, College of Medicine, King Saud University, Riyadh 12372, Saudi Arabia; 4 Department of Orthopedic Surgery, Security Forces Hospital, Ministry of Interior, Riyadh 12372, Saudi Arabia

**Keywords:** compartment syndrome, minor trauma, forearm, pediatrics

## Abstract

**Background and Clinical Significance:** Acute compartment syndrome is a rare but limb-threatening emergency in pediatric patients. While most cases follow high-energy trauma or displaced fractures, acute compartment syndrome precipitated by initially underestimated forearm injuries is uncommon and may create a significant diagnostic challenge, particularly in young children who exhibit atypical clinical presentations, such as escalating anxiety and analgesic requirements, rather than classic ischemic signs. **Case Presentation:** We report the case of a 4-year-old girl who developed severe forearm and hand compartment syndrome following a delayed presentation after a fall from a height of 2–2.5 m onto the left upper extremity. Initial evaluation revealed progressive tense swelling, severe pain with passive stretch, diminished distal perfusion, and radiographic evidence of distal radius-ulna buckle fractures associated with a proximal ulna fracture. Emergent surgical decompression via extensive volar and dorsal fasciotomies revealed markedly elevated compartment pressures. Intraoperatively, deep volar muscle ischemia and necrosis were identified, requiring carpal tunnel release, serial debridements, and complex staged wound management. Multidisciplinary care and ongoing rehabilitation were essential for limb salvage and functional recovery. **Conclusions:** This case underscores the profound unpredictability of pediatric compartment syndrome and demonstrates that even classically stable, benign fractures can initiate a devastating ischemic cascade. A high index of suspicion, regardless of the injury mechanism, along with early recognition and prompt surgical intervention, is absolutely critical for preventing irreversible myoneural damage and optimizing management outcomes in pediatric patients.

## 1. Introduction and Clinical Significance

Acute compartment syndrome (ACS) is a limb-threatening emergency that results from increased intracompartmental pressure leading to compromised tissue perfusion and ischemia [1]. If not recognized and treated promptly, sustained elevation of pressure within closed fascial compartments can rapidly lead to irreversible muscle necrosis, nerve injury, and permanent functional impairment. In pediatric patients, compartment syndrome of the forearm most commonly follows traumatic injuries, particularly fractures of the radius and ulna; however, its occurrence after minor or low-energy trauma is extremely rare, increasing the risk of delayed diagnosis [2]. Early diagnosis can be especially challenging in children due to atypical presentation, limited communication ability, and the absence of classic clinical findings traditionally described in adults.

Although ACS is less common in children, it remains a serious and potentially devastating condition with significant long-term morbidity when diagnosis is delayed. Reported cases are relatively rare, with trauma being the predominant etiology [3]. Among pediatric compartment syndromes, the forearm is one of the most frequently involved anatomical sites, accounting for 27% of cases [1]. The high incidence of forearm involvement is largely attributed to the prevalence of upper-extremity fractures in children, though cases without fracture or following minor trauma have also been reported [4,5]. In such situations, the seemingly benign nature of the initial injury may contribute to underestimation of severity by caregivers and healthcare providers, further increasing the likelihood of delayed recognition and intervention.

The pathophysiology of ACS involves a progressive cycle of increased compartmental pressure, reduced capillary perfusion, and worsening tissue hypoxia, ultimately resulting in myoneural ischemia. In pediatric patients, this process may evolve insidiously, as early warning signs are frequently subtle and nonspecific. Unlike adults, children often present with behavioral changes such as agitation, anxiety, or increasing analgesic requirements rather than the classic symptoms of pain out of proportion or paresthesia, making clinical assessment more complex. Consequently, a high index of suspicion is essential when evaluating pediatric patients presenting with progressive limb swelling following even apparently trivial injuries.

Delayed recognition of compartment syndrome can result in devastating complications, including irreversible muscle necrosis, nerve injury, infection, contracture formation, and long-term functional impairment [6]. The presence of vascular compromise, such as absent distal pulses or prolonged capillary refill, further complicates the clinical picture and necessitates urgent multidisciplinary intervention. Prompt surgical decompression through fasciotomy remains the cornerstone of treatment and is critical to salvage the limb and preserve long-term function [7]. Early intervention is particularly important in children, as timely decompression can significantly improve outcomes and reduce the need for complex reconstructive procedures.

We present the case of a 4-year-old child who developed severe forearm and hand compartment syndrome following a delayed presentation after forearm trauma, complicated by vascular compromise, muscle necrosis, and subsequent wound management challenges. This case highlights the importance of maintaining a high index of suspicion even after low-energy injuries, as well as the critical role of early recognition, multidisciplinary management, and staged surgical intervention in pediatric compartment syndrome. The patient’s guardian voluntarily agreed to publish this report for educational purposes, and informed consent was obtained.

## 2. Case Presentation

A previously healthy 4-year-old girl presented to the emergency department with progressive swelling and pain in the left forearm and hand following a fall from an estimated height of 2–2.5 m onto the left upper extremity one day prior to presentation. According to the patient’s caregivers, the child landed primarily on the elbow and wrist. However, over the subsequent day, the patient developed increasing pain, swelling, and decreased use of the affected limb, prompting medical evaluation.

**Figure 1:**
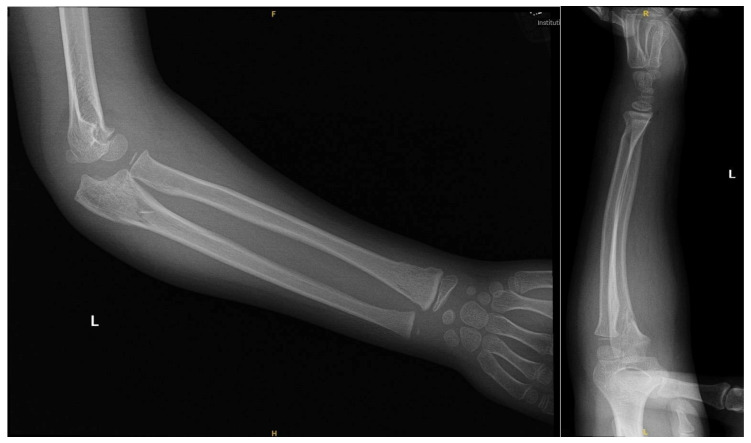
Anteroposterior and Lateral View of the Left Forearm Showing Distal Radius-Ulna Buckle Fracture along with Proximal Ulna Non-Displaced Fracture.

On examination, the affected forearm and hand were markedly swollen, tense, and tender to palpation. The child demonstrated significant pain with passive finger and wrist motion. The skin appeared taut with areas of discoloration, and distal capillary refill was delayed. Distal pulses were diminished on clinical examination, raising concern for vascular compromise. Initial radiographic evaluation revealed a buckle radius fracture with an associated non-displaced proximal ulna fracture (Figure 1). Given the clinical findings and delayed presentation, acute forearm and hand compartment syndrome was strongly suspected.

**Figure 2:**
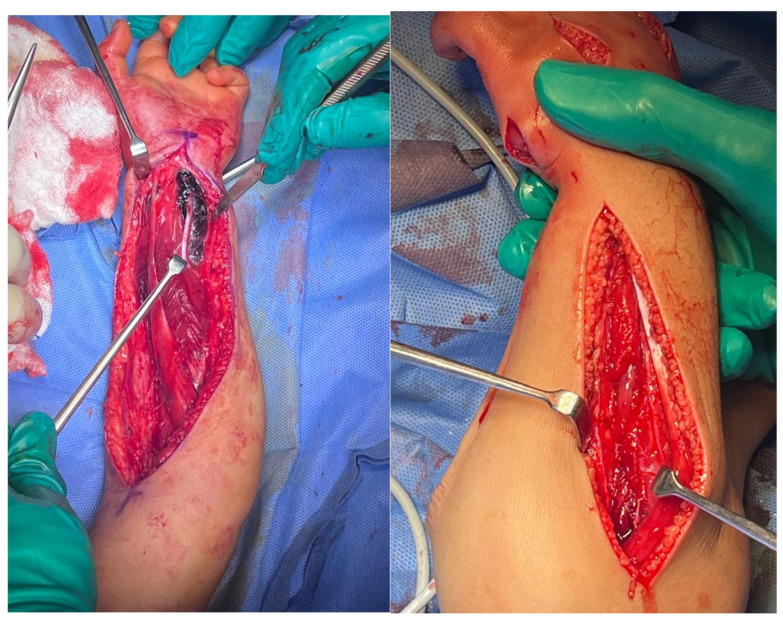
Intraoperative Images Demonstrating Volar and Dorsal Forearm Fasciotomy Incisions. Volar incision extended to include carpal tunnel release to relieve pressure over the median nerve. Necrotic deep volar muscles were identified, and nonviable areas were debrided.

**Figure 3:**
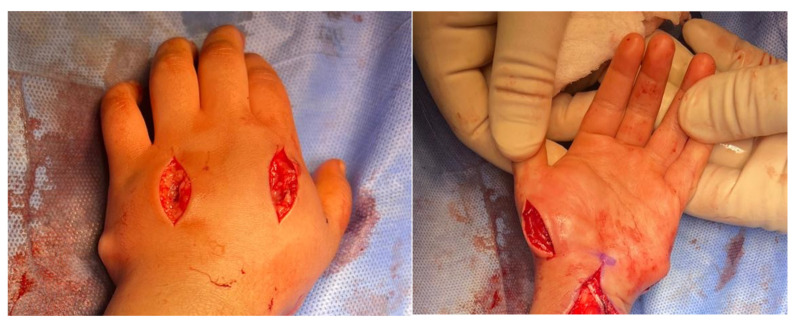
Intraoperative photographs of the hand fasciotomy incisions. Two dorsal incisions were made over the 2nd and 4th metacarpals. Two volar incisions were made over the Thenar and hypothenar muscles.

The patient was taken emergently to the operating room for surgical decompression. Extensive forearm and hand fasciotomies were performed. Intraoperatively, markedly elevated compartment pressures were encountered, and several muscle groups demonstrated signs of ischemia and necrosis (Figure 2 and Figure 3). Due to marked hand swelling, tense intrinsic compartments, delayed capillary refill, diminished distal perfusion, and absent Doppler signals at presentation, there was significant concern for concomitant hand compartment syndrome in addition to the forearm involvement. Intraoperatively, the hand remained markedly swollen and tense, further supporting decompression of the intrinsic hand compartments. Specific attention was given to complete decompression of all involved forearm and hand compartments. A standard volar forearm fasciotomy incision was performed extending from the proximal forearm across the wrist, with release of the flexor compartment fascia and extension into a formal carpal tunnel release to decompress the median nerve. A dorsal forearm fasciotomy was additionally performed to decompress the extensor compartment musculature. Due to marked hand swelling and concern for concomitant hand compartment syndrome, hand fasciotomies were also performed. Two dorsal longitudinal hand incisions were made over the second and fourth metacarpals to decompress the dorsal interosseous and adductor compartments, while separate volar incisions over the thenar and hypothenar eminences were used to decompress the intrinsic compartments of the hand. Intraoperatively, frank necrosis was identified predominantly within the flexor digitorum profundus (FDP) and pronator quadratus (PQ) muscles, while ischemic changes extended to portions of the flexor digitorum superficialis (FDS) and flexor pollicis longus (FPL) musculature. Non-viable muscle tissue was carefully debrided, and the wounds were left open to allow for continued decompression. Vascular status improved following fasciotomy, with restoration of distal perfusion and Doppler signals.

**Figure 4:**
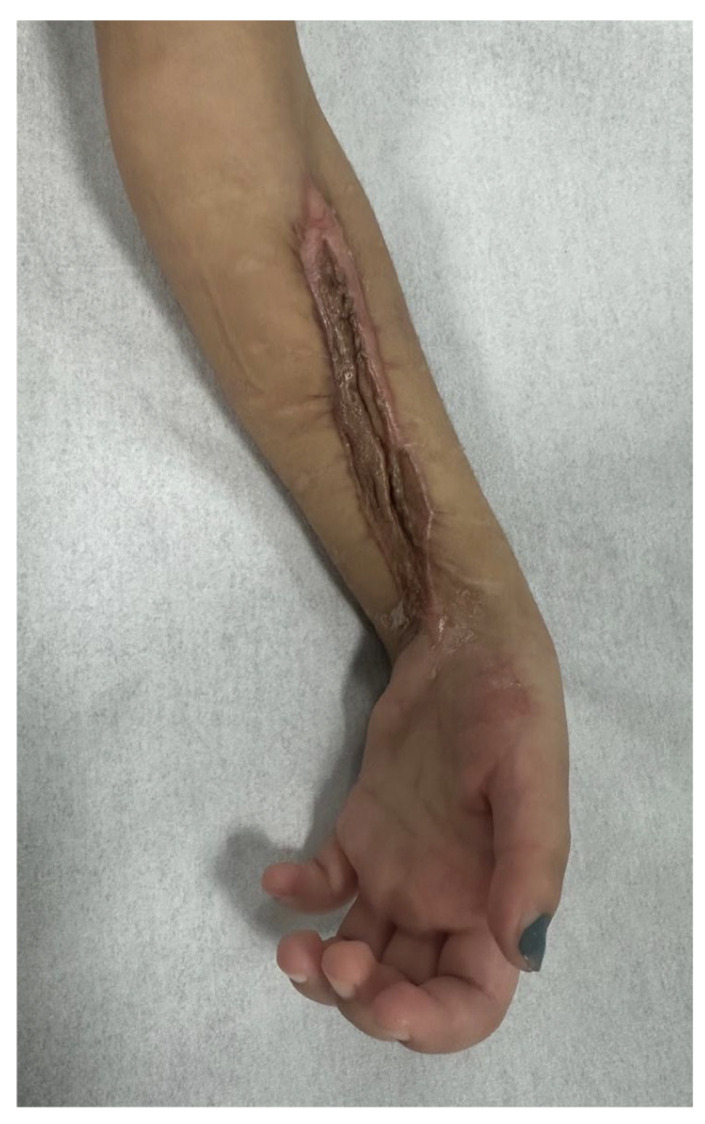
Latest post-closure image of the volar forearm wound.

Postoperatively, the patient was managed in a monitored setting with close neurovascular observation. Given the extent of soft tissue injury and muscle necrosis, staged surgical management was undertaken, including repeat debridements and meticulous wound assessments. The patient required prolonged wound care and multidisciplinary involvement, including pediatric orthopedics, plastic surgery, and rehabilitation services. Over the subsequent course, the wounds gradually stabilized, and definitive wound management was achieved once the soft tissues were deemed suitable (Figure 4). During the postoperative course, serial examinations demonstrated preserved limb vascularity with restoration of radial and ulnar Doppler signals and maintained digital perfusion. Early postoperative assessments demonstrated persistent limitation in finger movement consistent with the severity of the ischemic muscle injury. At follow-up, the patient demonstrated preserved limb viability with gradual improvement in function and remained enrolled in an ongoing rehabilitation program focused on recovery of hand and wrist function and overall functional use of the extremity.

## 3. Discussion

Acute compartment syndrome in pediatric patients is an uncommon but potentially devastating condition that requires a high index of suspicion for timely diagnosis. Compared with adults, children often present with atypical or subtle clinical findings, which can delay recognition and exponentially increase the risk of irreversible tissue damage. Although the injury in this case was initially underestimated, the patient ultimately demonstrated a significant forearm fracture pattern associated with progressive soft tissue compromise, vascular insufficiency, and extensive muscle ischemia. The delayed presentation to medical care likely contributed substantially to the severity of tissue injury encountered intraoperatively. Importantly, pediatric compartment syndrome often progresses more rapidly than expected because of the relatively smaller compartment volumes and limited physiologic reserve in young children, making early recognition even more critical for preventing irreversible ischemic injury. Furthermore, variability in symptom expression among young children frequently results in underestimation of disease severity during early evaluation, especially in cases with delayed presentation or evolving neurovascular compromise. The forearm is among the most commonly affected anatomical sites in pediatric compartment syndrome, mostly due to the high incidence of upper-extremity injuries in this active population [4]. Anatomically, the forearm contains tightly bound fascial compartments that have limited capacity to expand in response to accumulating edema or hemorrhage. However, arriving at a definitive diagnosis can be highly challenging, especially in younger children who may be unable to reliably communicate pain severity or pinpoint its exact location. In the present case, the mechanism of injury involved a fall from an estimated height of approximately 2–2.5 m onto the affected upper extremity. While no evidence of coagulopathy, constrictive casting, or pre-existing vascular abnormality was identified, the combination of multilevel forearm fractures, delayed presentation, progressive edema, and evolving vascular compromise likely contributed to the development of compartment syndrome. In addition, the presence of incomplete or minimally displaced fractures, such as buckle fractures, may falsely reassure clinicians despite their potential association with progressive soft tissue swelling and evolving compartment pressure elevation. This highlights the importance of correlating radiographic findings with serial clinical examination rather than relying solely on fracture morphology to estimate injury severity. Several reports in the literature have described ACS developing after initially being underestimated, emphasizing the potential discordance between fracture appearance, reported mechanism, and the subsequent severity of soft tissue injury [5,8,9]. Everaert et al. described an atypical pediatric forearm compartment syndrome following minor trauma in which delayed recognition contributed to progressive ischemic injury despite initially non-alarming clinical findings [5]. Similarly, Kanj et al. reported that delayed diagnosis remains common in pediatric upper-extremity compartment syndrome because children frequently present with nonspecific symptoms and evolving examination findings rather than classic adult manifestations [2]. These reports collectively highlight that compartment syndrome in children should not be excluded solely on the basis of apparently stable fracture morphology or an initially underestimated mechanism of injury. In the present case, several factors likely contributed synergistically to the development and progression of compartment syndrome, including the multilevel forearm fracture pattern, delayed presentation, progressive edema, and evolving vascular compromise. Importantly, no evidence of constrictive casting, coagulopathy, thrombocytopenia, anticoagulant exposure, or underlying vascular abnormality was identified during the patient’s evaluation or operative management. Furthermore, although no definitive arterial injury was observed intraoperatively, the markedly diminished distal perfusion and absent Doppler signals at presentation suggest that progressive intracompartmental pressure elevation had already resulted in significant vascular compromise prior to surgical decompression. These findings further emphasize the importance of repeated clinical reassessment and early surgical intervention in pediatric patients with evolving swelling and neurovascular deterioration following forearm trauma [8,9]. While the classic “six Ps” pain out of proportion, paresthesia, pallor, paralysis or motor weakness, pulselessness, and poikilothermia remain commonly used diagnostic criteria for compartment syndrome in adults, their reliability in children is heavily limited. Expecting a toddler or preschooler to accurately describe paresthesia is unrealistic, and waiting for paralysis or pulselessness guarantees poor management outcomes. Consequently, the pediatric orthopedic community has shifted toward relying on a pediatric-specific mnemonic known as the “three As”: increasing Anxiety, Agitation, and Analgesic requirements [10,11,12]. In a child with a known or suspected extremity injury, unremitting agitation that does not respond to appropriate weight-based analgesia should immediately trigger a strong suspicion of ACS. Recognition of these behavioral indicators is particularly valuable in emergency settings, where rapid clinical decisions must often be made before advanced diagnostic adjuncts can be obtained. Furthermore, the utility of objective compartment pressure monitoring in pediatric patients remains debated. While intracompartmental pressure measuring devices are invaluable in unconscious or polytrauma patients, obtaining accurate readings in an awake, terrified child is technically difficult and can cause further distress, potentially yielding artificially elevated pressure readings due to crying and muscle contraction. Therefore, the diagnosis of pediatric acute compartment syndrome remains primarily clinical. Serial bedside reassessment by experienced clinicians remains one of the most reliable strategies for detecting evolving compartment syndrome in equivocal cases. Close observation over short intervals is especially important when symptoms appear disproportionate to the overall clinical presentation and evolving neurovascular findings. Delayed presentation remains one of the most significant and well-documented risk factors for poor outcomes in pediatric compartment syndrome [13]. Prolonged elevation of intracompartmental pressures initiates a catastrophic cascade that can quickly lead to muscle ischemia, necrosis, nerve injury, and vascular compromise, as was clearly observed in the present case [14]. The ischemic threshold for skeletal muscle is widely variable, but significant irreversible damage can occur within hours of the onset of critical pressure levels. The presence of diminished pulses and delayed capillary refill in our patient indicated highly advanced disease and necessitated urgent surgical intervention [15]. Importantly, the medical community must continually emphasize that the absence of pulses is a late and ominous finding; it represents an extreme state of ischemia and should absolutely not be awaited before proceeding with a fasciotomy. Even partial vascular compromise should therefore be interpreted as a surgical emergency requiring immediate decompression to prevent progression to complete ischemia. In our patient, the delayed presentation likely contributed directly to the extent of muscle necrosis encountered intraoperatively, emphasizing the narrow therapeutic window available for limb salvage in pediatric cases. Intraoperatively, frank necrosis predominantly involved the FDP and PQ muscles, while ischemic changes extended into portions of the FDS and FPL musculature. Prompt surgical decompression through complete fasciotomy remains the only definitive treatment for compartment syndrome. Incomplete release of the fascial envelopes is a common pitfall that can lead to continued ischemia despite surgical intervention. In pediatric patients with delayed presentation and extensive soft tissue injury, a single surgical procedure is rarely sufficient; a staged surgical approach is often required. This methodology includes initial emergent decompression, followed by serial debridements in the operating room to address evolving muscle necrosis, and careful, continuous wound management [16]. Such staged operative strategies also permit reassessment of marginally viable muscle groups that may initially appear ischemic but recover following reperfusion. This approach helps optimize tissue preservation while minimizing unnecessary excision of potentially recoverable musculature. The management of the open fasciotomy wound also presents a unique challenge in the pediatric population. Techniques such as negative pressure wound therapy (NPWT) or the use of dynamic wound closure devices are often employed to manage exudate, reduce edema, and gradually re-approximate the skin edges. Multidisciplinary involvement, including pediatric orthopedics, plastic surgery, and specialized pediatric rehabilitation services, is absolutely essential to optimize soft tissue coverage and ensure maximal functional recovery [17]. Rehabilitation must focus not only on regaining range of motion and strength but also on addressing the psychological impact of the trauma and subsequent prolonged hospital course. Early collaboration with rehabilitation specialists is particularly important in young children to prevent stiffness-related functional loss during critical developmental stages of motor recovery. Structured follow-up programs further support long-term monitoring for late complications such as contracture formation or growth-related deformity. This case underscores several vital clinical lessons. First, compartment syndrome must be considered in the differential diagnosis even after forearm trauma in young children presenting with progressive limb pain, out-of-proportion swelling, and increasing analgesic requirements. Second, delayed presentation significantly increases the risk of extensive muscle necrosis and long-term morbidity, emphasizing the need for robust parental education regarding the warning signs of vascular compromise after any extremity injury. Finally, early clinical recognition, urgent surgical decompression without relying solely on late vascular signs, and highly coordinated multidisciplinary care are critical to achieving limb salvage and functional preservation in pediatric compartment syndrome. Taken together, this case emphasizes the importance of maintaining a high index of suspicion for evolving compartment syndrome in pediatric forearm injuries, particularly in the setting of delayed presentation, progressive swelling, disproportionate pain, and neurovascular compromise [5]. Early recognition, repeated clinical assessment, and timely surgical decompression remain essential to minimize irreversible myoneural injury and optimize long-term functional outcomes. Awareness of these atypical presentations may help reduce diagnostic delay and improve long-term functional outcomes in similar cases reported in future clinical practice.

## 4. Conclusions

This case underscores the unpredictability of pediatric compartment syndrome and the critical need for a high index of suspicion regardless of the injury mechanism or initial radiographic findings. Delayed presentation following forearm trauma may result in progressive soft tissue compromise, vascular insufficiency, and severe compartment syndrome in young children. Healthcare providers evaluating pediatric extremity injuries must remain vigilant for atypical presentations, relying on clinical signs such as disproportionate agitation and escalating analgesic needs. Early recognition and prompt, definitive surgical decompression remain the most critical factors in preventing irreversible tissue damage and ensuring the best possible functional outcomes for these vulnerable patients. Furthermore, this case highlights the importance of caregiver education regarding warning signs after pediatric extremity injuries and reinforces the value of early multidisciplinary involvement to optimize limb salvage, guide staged wound management, and support long-term functional rehabilitation in pediatric patients with advanced compartment syndrome.

## Data Availability

The original data presented in the study are included in the article. Further inquiries can be directed to the corresponding authors.

## Abbreviations

The following abbreviations are used in this manuscript:

ACS	Acute compartment syndrome
FDP	Flexor digitorum profundus
PQ	Pronator quadratus
FDS	Flexor digitorum superficialis
FPL	Flexor pollicis longus
